# FISH-negative, cytogenetically cryptic acute promyelocytic leukemia

**DOI:** 10.1038/bcj.2015.47

**Published:** 2015-06-19

**Authors:** A Rashidi, S I Fisher

**Affiliations:** 1 Division of Oncology, Washington University School of Medicine, St Louis, MO, USA; 2 Pathology Sciences Medical Group/Eastern Virginia Medical School, Norfolk, VA, USA

Approximately 600–800 new cases of acute promyelocytic leukemia (APL) occur annually in the United States.^1^ The advent of all-*trans* retinoic acid (ATRA) has converted this subtype of acute leukemia to a readily curable one with excellent long-term outcomes. Rapid diagnosis and immediate treatment is crucial in APL and a requirement for favorable prognosis. t(15;17)(q24;q21) is the characteristic translocation and the defining feature of APL, resulting in reciprocal *PML*-*RARA* translocation in 98% of cases.^2^ Although immediate initiation of ATRA upon morphological and clinical suspicion of APL is recommended to prevent the typical and often devastating bleeding diathesis associated with APL, definitive diagnosis relies on the demonstration of *PML*-*RARA* translocation. Cytogenetically cryptic APL, without t(15;17)(q24;q21) on conventional chromosomal analysis, is known to occur, albeit rarely. These cases are usually due to a cryptic insertion of the *RARA* gene into the *PML* gene or vice versa, without the reciprocal *RARA*/*PML* fusion.^3^ Such genetic derangements are usually, but not always, detectable by fluorescence *in situ* hybridization (FISH; including a *RARA* break-apart probe), especially using smaller probes.^4^ Unfortunately, however, rare cases of APL have been reported with normal karyotype and negative FISH. Although the diagnosis in such cases can be established by reverse transcriptase PCR (RT-PCR), the clinical challenge in FISH-negative, cytogenetically cryptic APL is in making a decision on continuation of ATRA before the diagnosis is definitively established.

Three isoforms of *PML*-*RARA* have been identified:^5, 6^ (i) short isoform (S; bcr-3) due to a breakpoint in *PML* (intron 3) resulting in fusion of *RARA* (exon 3) with *PML* (exon 3); (ii) long isoform (L; bcr-1) due to a breakpoint in *PML* (intron 6) resulting in fusion of *RARA* (exon 3) with *PML* (exon 6); and (iii) variant isoform (V; bcr-2) due to a breakpoint in *PML* (exon 6) resulting in fusion of *RARA* (exon 3) with *PML* (exon 6). The breakpoint in *RARA* is invariably located in intron 2. Isoforms bcr-3, bcr-1 and bcr-2 occur in ~40–45%, 45–55% and 10% of patients with APL, respectively. Herein we review all previously reported cases of cytogenetically cryptic, FISH (interphase and metaphase)-negative APL. We used keywords ‘acute promyelocytic leukemia', ‘cryptic' and ‘ins(15;17)' for our PubMed search and reviewed all relevant references in the extracted reports. Rare *NPM-RARA* and *NuMA-RARA* variants, as well as ATRA-resistant variants such as *PLZF*-*RARA* and *STAT5b*-*RARA* cases, were not included in this review.

A total of 23 eligible cases are included (Table 1 and Supplementary Information). Clues to diagnosis were based on highly characteristic morphological, immunophenotypical and clinical features. The median (range) age was 39 (14–72) years and 50% were male. None of the patients were classified as secondary or therapy-related. Laboratory and/or clinical evidence of disseminated intravascular coagulation (DIC) were present at presentation in 85% of patients. Median (range) white blood cells (WBCs), hemoglobin and platelets at presentation was 10.0 (0.6–242) × 10^9^/l, 8.9 (6.6–12.6) g/dl and 36 (9–155) × 10^9^/l, respectively. In all, 70% and 61% of patients were anemic and thrombocytopenic at presentation, respectively. According to the current risk stratification scheme,^7^ 44% of patients were high risk (WBC>10.0 × 10^9^/l). The median (range) blast percentage in the marrow was 89 (61–95)%. Karyotypic abnormalities other than t(15;17) were present in 52% of patients. Although this rate appears higher than those reported for non-cryptic APL (30%),^8, 9^ we are underpowered for statistical analysis. Also, trisomy 8 was the most common abnormality in our cases (25%), similar to non-cryptic APL reports.^8, 9^ The specific PML-RARA isoform was not reported in two reports, and the diagnosis of APL was established on the basis of morphology and panel's consensus despite negative RT-PCR in three cases. In all other cases, a PML-RARA transcript was detected using RT-PCR. The detected isoform was bcr-3 (S) in 61% and bcr-1 (L) in the remainder. Induction was ATRA based (with or without chemotherapy) in 70% of patients, arsenic trioxide (ATO) alone in 10% and chemotherapy alone in 10%. The remaining 10% died before treatment was initiated. Early death (ED; within 30 days of hospitalization) occurred in 3 (18%) of the 17 patients for whom outcome data were available. One of these patients received ATRA-based induction and the other two died before treatment initiation. All patients who achieved remission with induction remained in remission during the follow-up period (median: 6 months; maximum: 24 months). Numbers were too small to perform detailed survival analysis, but the ED rate in this series appears similar to those reported for non-cryptic APL patients.^10, 11^

In conclusion, FISH-negative, cytogenetically cryptic APL is an exceedingly rare but important diagnosis. Failure to make the correct diagnosis can have a catastrophic outcome. This is particularly important given our observation that 85% of patients had laboratory and/or clinical evidence of DIC at presentation. The majority, if not all, of such cases can be diagnosed using RT-PCR, which remains the most efficient diagnostic modality. Translocation-based comparative genomic hybridization, a modified version of genomic microarray analysis developed to detect balanced translocations using a linear amplification of a potential translocation breakpoint region in a genomic DNA specimen, is another potentially useful strategy.^12^ Sanger and whole-genome sequencing are other methods that can be used in challenging cases.^13, 14^ Recognition of suggestive morphology and typical clinical and laboratory findings in FISH-negative, cytogenetically cryptic cases and immediate initiation of ATRA followed by its continuation until RT-PCR results are available are key to successful treatment. A remaining challenge in such cases is to decide on what agent(s) to add to ATRA while awaiting the final diagnosis. Considering that chemotherapy is not needed in patients with low-risk APL (WBC<10.0 × 10^9^/l),^15^ we recommend using a combination of ATRA and ATO if morphologically and clinically suspicious cases have a low WBC count. On the other hand, we favor ATRA along with chemotherapy in patients who would be at high risk (WBC>10.0 × 10^9^/l) if diagnosed with APL. Our results from this review suggest ATRA/ATO underutilization (10% of patients received chemotherapy alone) and demonstrate the need for improvement in clinical and morphological diagnosis of APL.

## Figures and Tables

**Table 1:** Summary of previous cases of FISH-negative, cytogenetically cryptic APL

*Ref*^a^*/Age/Sex/DIC/WBC/Hb/Plt/BM blasts*	*Karyotypic abnormalities*	*Isoform on RT-PCR*	*Induction* *Outcome*
3/NA/M/NA/NA/NA/NA/NA	der(7)t(7;11)(q34;p15), der(11)t(7;11), ins(7;12)(q34;q24.3), −Y	None^b^	NA
3/NA/M/NA/NA/NA/NA/NA	Normal	None^b^	NA
3/NA/M/NA/NA/NA/NA/NA	Normal	None^b^	NA
12/57/M/NA/6.8/8.9/40/83%	Normal	L	ATRA+Chemo CR 5 m
13/17/M/+/9.9/NA/NA/95%	Normal	L	ATRA+ATO+Chemo CR 2y
14/39/F/+/1.3/11.6/72/61%	del(9)(q12q32), del(12)(q12q21), −6, −16, add(16)(p13.2), +2 mar	S	ATRA+Chemo CR 15 m
S1/29/M/NA/L/L/L/73%	Normal	L	ATRA+chemo NA
S2/24/M/+/64.3/NA/NA/95%	Normal	NA	ATRA+ATO+Chemo CR 2 m
S3/72/M/+/20.4/10.3/22/88%	t(3;17)(p25;q21), +8	S	None Early death
S4/46/M/+/1.7/10.6/18/NA	92, XXYY	S	ATRA+Chemo CR 14 m
S5/18/M/NA/16.5/9.5/37/94%	Normal	L	ATRA NA
S6/30/F/+/242.2/8.8/20/NA	t(9;22)(q34;q11.2)	S	Chemotherapy CR 3 m
S7/48/F/+/14.9/8.8/36/84%	+8	S	ATRA+Chemo CR 1 m
S8/39/F/+/16.9/7.6/17/95%	+7q	S	ATO CR 1 m
S9/25/F/+/2.9/8.3/9/95%	−9q22	L	ATRA+Chemo Early death
S10/26/F/−/0.6/7.7/155/86%	−5q	S	ATRA+Chemo CR 8 m
S10/33/F/−/1.4/7.1/10/90%	Normal	S	ATO CR 12 m
S10/46/M/+/63.8/6.6/146/71%	−5, −12q24.1, −19p13	L	NA
S11/63/F/NA/ NA/NA/NA/NA	Normal	S	Chemo CR 6 m
S11/14/M/ NA/ NA/NA/NA/NA	Normal	S	ATRA+Chemo CR 10 m
S12/44/F/NA/1.5/12.6/49/NA	i(17)(q10)	L	ATRA+Chemo CR
S13/50/F/NA/101.7/9.9/16/NA	+8	S	ATRA+Chemo CR 5 m
S14/67/M/+/1.9/10.8/89/95%	Normal	NA	Early death

Abbreviations: ATO, arsenic trioxide; ATRA, all-*trans* retinoic acid; BM, bone marrow; CR, complete remission; DIC, disseminated intravascular coagulation; F, female; Hb, hemoglobin; L, long isoform (bcr-1); m, month; M, male; NA, not available in the original report; RT-PCR, reverse transcriptase PCR; Plt, platelets; S, short isoform (bcr-3); WBC, white blood cell; y, year.

a References identified by prefix ‘S' are provided in Supplementary Information.

b Morphologic diagnosis and according to panel's consensus despite a negative RT-PCR.
