# Supplementary material for: FISH-negative, cytogenetically cryptic acute promyelocytic leukemia
Source: Blood Cancer J. 2015 Jun 19;5(6):e320–. doi: 10.1038/bcj.2015.47 (PMC4648483; doi:10.1038/bcj.2015.47)
Supplement: Supplementary Information [file bcj201547x1.doc]

**Supplementary information:** Additional references cited in Table 1.

**Supplementary references**

1. Koshy J, Qian YW, Bhagwath G, Willis M, Kelley TW, Papenhausen P. Microarray, gene sequencing, and reverse transcriptase-polymerase chain reactionanalyses of a cryptic PML-RARA translocation. *Cancer Genet* 2012; **205:** 537-540.
2. Lewis C, Patel V, Abhyankar S, Zhang D, Ketterling RP, McClure RF, *et al*. Microgranular variant of acute promyelocytic leukemia with normal conventional cytogenetis, negative PML/RARA FISH and positive PML/RARA transcripts by RT-PCR. *Cancer Genet* 2011; **204:** 522-523.
3. Chattopadhyay A, Redner RL. Cryptic insertion of PML-RARA into the 3p25 locus in an acute promyelocytic leukemia with t(3;17)(p25;q21). *Cancer Genet Cytogenet* 2010; **201:** 28-31.
4. Soriani S, Cesana C, Farioli R, Scarpati B, Mancini V, Nosari A. PML/RAR-alpha fusion transcript and polyploidy in acute promyelocytic leukemia withoutt(15;17). *Leuk Res* 2010; **34:** 261-263.
5. Kim MJ, Cho SY, Kim MH, Lee JJ, Kang SY, Cho EH, *et al*. FISH-negative cryptic PML-RARA rearrangement detected by long-distance polymerasechain reaction and sequencing analyses: a case study and review of the literature. Cancer Genet Cytogenet 2010; **203:** 278-283.
6. Emilia G, Marasca R, Longo G, Ferrari MG, Notohamiprodjo M, Temperani P, *et al*. Detection of PML-RAR alpha fusion transcript in Ph positive leukemia with acute promyelocytic phenotype lacking the t(15;17) cytogenetic abnormality. *Cancer Genet Cytogenet* 1995; **80:** 95-99.
7. Han JY, Kim KE, Kim KH, Park JI, Kim JS. Identification of PML-RARA rearrangement by RT-PCR and sequencing in an acute promyelocytic leukemia without t(15;17) on G-banding and FISH. *Leuk Res* 2007; **31:** 239-243.
8. Wang Y, Fang M, Jing Y, Li J, Jiang F, Wang Y. Derivative (7)t(7;8): The sole karyotype abnormality in acute promyelocytic leukemia withPML/RARA rearrangement identified by RT-PCR and sequence analysis. *Leuk Res* 2009; **33:** 55-58.
9. Yamamoto K, Hamaguchi H, Kobayashi M, Tsurukubo Y, Nagata K. Terminal deletion of the long arm of chromosome 9 in acute promyelocytic leukemia with a cryptic PML/RAR alpha rearrangement. Cancer Genet Cytogenet 1999; **113:** 120-125.
10. Choughule A, Polampalli S, Amre P, Shinde S, Banavali S, Prabhash K, *et al*. Identification of PML/RARalpha fusion gene transcripts that showed no t(15;17) with conventional karyotyping and fluorescent in situ hybridization. *Genet Mol Res* 2009; **8:** 1-7.
11. Kim M, Lim J, Kim Y, Han K, Lee DH, Chung NG, *et al*. The genetic characterization of acute promyelocytic leukemia with cryptic t(15;17) including a new recurrent additional cytogenetic abnormality i(17)(q10). *Leukemia* 2008; **22:** 881-883.
12. Huh J, Moon H, Chi H, Chung W. Acute promyelocytic leukemia with i(17)(q10) on G-banding and PML/RARA rearrangement byRT-PCR without evidence of PML/RARA rearrangement on FISH. *Int J Lab Hematol* 2009; **31:** 372-374.
13. Kim KE, Woo KS, Kim SH, Han JY. [Detection of PML/RARA rearrangement by reverse transcriptase-PCR and sequencing in a case of microgranular acute promyelocytic leukemia lacking t(15;17) on karyotype and FISH]. *Korean J Lab Med* 2009; **29:** 379-383.
14. Rashidi A, Fisher SI. FISH: negative. Morphology: positive. Blood 2014; **124:** 3501.
